# Causal relationships between 91 inflammatory cytokines and gastroesophageal reflux disease, and the mediating role of related metabolites: Evidence from genetics

**DOI:** 10.1097/MD.0000000000042426

**Published:** 2025-05-30

**Authors:** Liqun Li, Lijian Liu, Jieru Xie, Chengning Yang, Guangwen Chen, Jinjing Tan, Xiaoyan Huang, Sheng Xie

**Affiliations:** a Department of Gastroenterology, The First Affiliated Hospital of Guangxi University of Chinese Medicine, Nanning, Guangxi, China.

**Keywords:** bidirectional Mendelian randomization analysis, circulating inflammatory cytokines, Mendelian randomization, gastroesophageal reflux disease

## Abstract

The associations between specific inflammatory cytokines and gastroesophageal reflux disease (GERD) have been confirmed, while their causal relationships remain unclear. We conducted a bidirectional 2-sample Mendelian randomization analysis (MRA) to reveal the causal correlations between 91 inflammatory factors and GERD, thereby conducting a 2-step MRA analysis to reveal the mediating function of 1400 metabolites therein. Data related to 91 inflammatory cytokines were derived from Genome-Wide Association Studies (GWAS) data with 14,824 samples. GWAS data about GERD were obtained from a large genome research project in Finland, including 28,859 GERD cases and 3,50,064 controls. GWAS data about 1400 circulating metabolites were derived from a cohort of 8299 individuals. The inverse variance weighted approach was introduced to obtain the primary MRA results, complemented by 4 additional methods for supportive analyses. Additionally, the study conducted sensitivity analyses with different methods to evaluate heterogeneity and horizontal pleiotropy. Our findings indicated that higher predicted C-X-C motif chemokine 1 [OR = 1.052, 95% CI: 1.004 − 1.103, *P* = .035], interleukin-1-alpha [OR = 1.083, 95% CI: 1.018 − 1.152, *P* = .012], and interleukin-4 [IL-4, OR = 1.081, 95% CI: 1.018 − 1.147, *P* = .011] increased the risk of GERD; whereas tumor necrosis factor ligand superfamily member 12 [OR = 0.958, 95% CI: 0.9174–0.9999, *P* = .0493] decreased the risk of GERD. No significant statistical differences were found for other inflammatory cytokines. Genetic susceptibility to GERD had no impact on any traits related to circulating inflammatory cytokines. Four metabolites associated with elevated IL-4 levels were identified, including (1-enyl-oleoyl)-GPE (*P*-18:1), 4-methyl guaiacol sulfate, X-12730, and X-17685, but their mediating effects were not statistically significant (*P* > .05). This study convincingly proved the causal impact of inflammatory factors on GERD. It confirms that C-X-C motif chemokine 1, interleukin-1-alpha, and IL-4 increase the risk of GERD, while tumor necrosis factor ligand superfamily member 12 reduces it. Additionally, several circulating metabolites are associated with elevated IL-4 levels. However, the evidence for their role as potential mediators in the pathway between IL-4 and GERD risk remains insufficient. The findings of this study propel further comprehension of the pathogenesis of GERD and also point out the therapeutic potential for interventions targeting inflammatory cytokine-targeted inventions.

## 1. Introduction

Gastroesophageal reflux disease (GERD) is a condition that gastric contents refluxing into the esophagus, mainly manifests as acid regurgitation and heartburn and is often accompanied by extra-esophageal symptoms, e.g. chronic cough, hoarseness, asthma, pharyngitis, and palpitations.^[1]^ Epidemiological data indicates a global prevalence of approximately 13.98%, with rates in some regions as high as 27.5%.^[2]^ Over the past 2 decades, there has been a 74.79% increase in total morbidity and a 77.19% increase in mortality related to GERD.^[3]^ Currently, due to the unclear pathogenesis of GERD, clinical efficacy remains unsatisfactory. Proton pump inhibitors are able to relieve the symptoms effectively, however, more than half (57.29%) of patients suffer from relapse after treatment, along with multiple adverse effects.^[4]^ Chronic and refractory GERD can increase the risk of coronary heart disease, esophageal cancer, lung cancer, and interstitial pneumonia, which can have a huge impact on the general health of the patients.^[5]^ Thus, promoting understanding of GERD mechanisms and improving clinical strategies are crucial for developing more effective prevention and treatment methods.

The exact mechanisms of GERD are, by far, still elusive. However, the complex interactions between GERD and inflammatory cytokines have already been revealed. Evidence indicates that esophageal acid exposure is directly associated with the expression of genes for tumor necrosis factor-a, interleukin-10 (IL-10) and interleukin-8 (IL-8).^[6]^ The progression of GERD may enhance the expression of various inflammatory mediators, for instance IL-8, interleukin-6 and IL-10 expressed.^[7]^ However, controversy continues on whether inflammatory cytokines are the cause or a consequence of GERD progression. Although evidence derived from observational studies has interpreted the causal association between inflammatory cytokines and GERD, the results can be biased. The main causes of biased results are the inability of observational studies to exclude potential confounders or the presence of reverse causality. Therefore, their causality still remains unclear. A reliable causal inference method then turns out necessary to clarify the causal correlations between inflammatory factors and GERD.

Mendelian randomization analysis (MRA) studies utilize genetic instrumental variables (IVs) to determine causal associations between modifiable exposures and outcomes, thereby functioning as “natural” randomized controlled trials.^[8,9]^ MRA is principled on Mendel’s laws of inheritance.^[9]^ MRA is not only effective in avoiding the effects of confounding factors present in conventional observational epidemiological studies but also excludes possible reverse causality.^[9]^ Although its widespread application in the exploration of risk factors, as far as it is concerned, no research has yet explored the associations between inflammatory cytokines and GERD, nor the potential pathway, especially for circulating metabolites via this method. Utilizing bidirectional 2-sample MRA, the study then aims to hopefully fill the gap by revealing the associations between circulating inflammatory factors and GERD, as well as using 2-step MRA to explain whether circulating metabolites are potential mediators therein, and finally offering a positive contribution to the management of GERD.

## 2. Materials and methods

### 2.1. Study design and assumption

**Figure 1.**
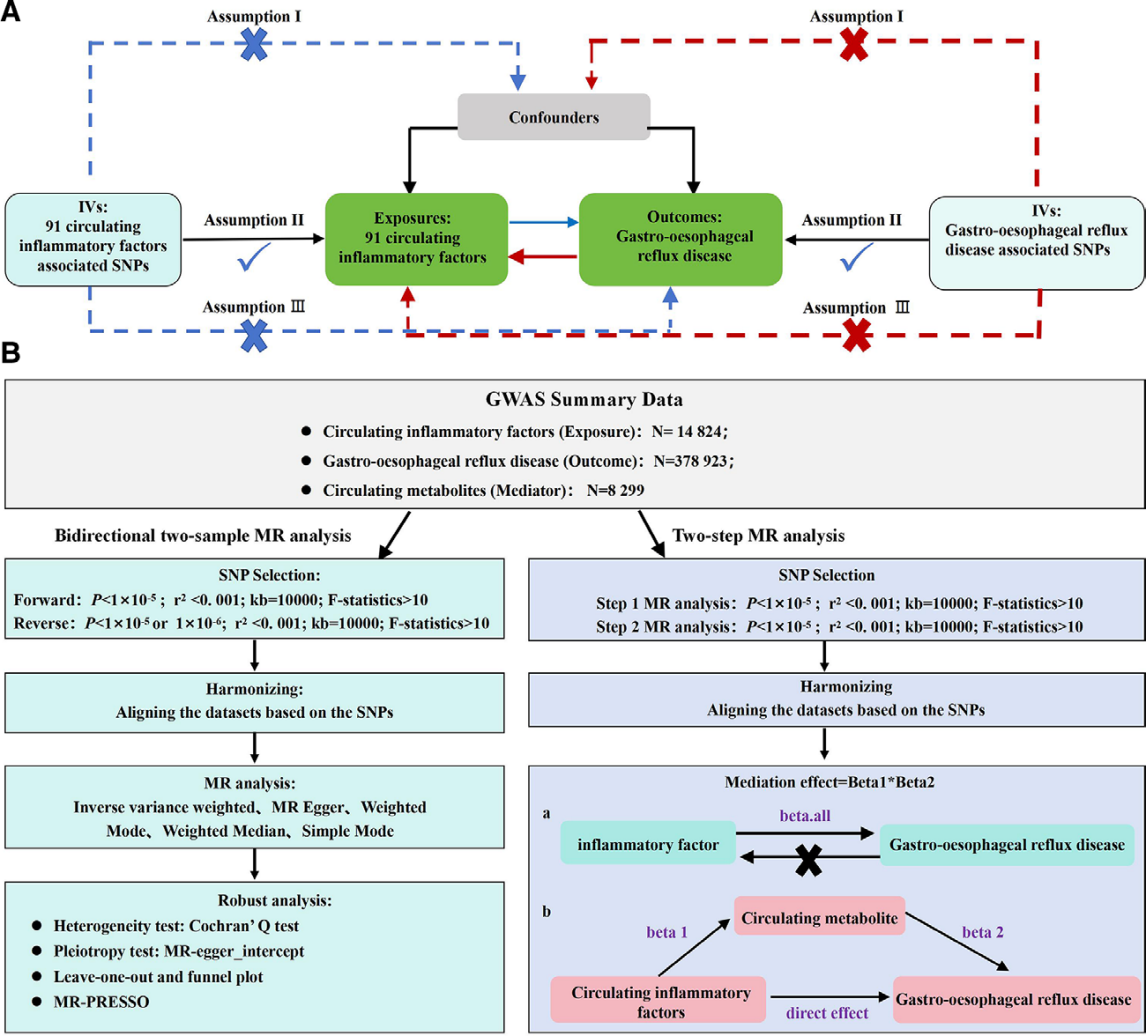
(A) Schematic diagram of Mendelian randomization principles. (B) Flow chart of 2-way 2-sample MR analysis. MR = Mendelian randomization.

There are 3 defining assumptions in this study: The genetic variants must be strongly associated with exposure. The genetic variants is not associated with confounding factors without any pleiotropic association. The genetic variants influence outcome solely through exposure. The study utilized the publicly available genome-wide association studies (GWAS) data for the association of 91 inflammation cytokines and GERD. First, we inferred the causal associations of 91 inflammatory cytokines on GERD. Next, the causal associations of GERD on each circulating inflammatory cytokines was inferred. Finally, mediation analysis was performed on 1400 blood metabolites to infer the mediating role of metabolites therein. As the study relied on public genetic data, no ethical approval was necessary. The study design and manuscript followed the guidelines of *Strengthening the Reporting of Observational Studies in Epidemiology-Mendelian randomization*.^[10]^ The study design is illustrated in Figure 1.

### 2.2. GWAS data sources

The GWAS data for 91 inflammatory cytokines were collected from 11 cohorts, involving 14,824 European individuals (Table S1, Supplemental Digital Content, https://links.lww.com/MD/O970).^[11]^ The complete GWAS data for this study are available on the website of the Department of Public Health and Primary Care, University of Cambridge (https://www.ebi.ac.uk/gwas/, Accession No. GCST90274758 to GCST90274848). A detailed explanation of data management can be found in the original publication.^[11]^

The GWAS data for GERD was downloaded in FinnGen, a large genome research project in Finland.^[12]^ Initiated in 2017, FinnGen is one of the largest genome research projects aimed at improving human health through genetic research and ultimately identifying new therapeutic targets and diagnostic methods for various diseases. The GWAS data ID for GERD is“finngen_R10_K11_REFLUX.gz.” This study included a total of 378,923 European participants (Table S2, Supplemental Digital Content, https://links.lww.com/MD/O970).

Data on 1400 circulating metabolites were derived from a cohort of 8299 individuals in GWAS,^[13]^ and the complete summary data is available on the NHGRI-EBI GWAS Catalog (https://www.ebi.ac.uk/gwas/, Table S3, Supplemental Digital Content, https://links.lww.com/MD/O970).

### 2.3. Genetic instrumental variable selection

First, the IVs selected for analysis must have strong associations with the exposure. To ensure involved IVs are adequate for screening, the condition for single nucleotide polymorphisms (SNPs), related to inflammatory factors and metabolites, was set to *P* < 1e-5. Initially, we screened GERD as exposed SNPs with a screening condition of *P* < 1e-5, but when there was pleiotropy in the statistics, we adjusted its screening pick to *P* < 1e-6. And the involved IVs need to meet the requirements of the independence test. To examine the independence and linkage disequilibrium effects of these variables, parameters for removing linkage disequilibrium were set to 10,000 kb and *r*^2^ = 0.001. Finally, the strength of each SNP was assessed using the *F*-statistic (*F* > 10). The Detailed information on SNPs analyzed in our MRA can be found in Table S1–S3, Supplemental Digital Content, https://links.lww.com/MD/O970.

### 2.4. Bidirectional Mendelian randomization analysis

This study conducted a bidirectional 2-sample MR analysis to assess the causal associations between 91 inflammatory cytokines and GERD. The inverse variance weighted (IVW) approach was implemented for preliminary analysis to obtain MRA predictive values, which is a robust method.^[14]^ MR-Egger, Weighted Mode, Weighted Median,^[15]^ and Simple Mode were used for secondary analysis. The pleiotropy was identified by using MR-Egger intercept.^[16]^ Heterogeneity was calculated by employing Cochran’s Q test with a condition that *P* > .05 suggested a low possibility of heterogeneity.^[17]^ If heterogeneity existed, random-effects IVW would be adopted for the primary analysis. Utilizing Mendelian randomization pleiotropy residual sum and outlier (MR-PRESSO), outliers were identified. if any was identified, then the analysis would be repeated and rectify pleiotropy.^[18]^ Employing sensitivity analysis to test the stability of the results. A significance level of *P* < .05 indicated a noteworthy association. The data underwent analysis using the 2-Sample MRA and software packages within R software (version R 4.3.3).

### 2.5. Mediation analysis

We proceeded with mediation analysis for 1400 metabolites using a 2-step MRA approach. The overall effect consists of indirect effects (mediation-indicated) and direct effects (mediation-indicated).^[19]^ First, MRA for inflammatory cytokines and metabolites was performed to obtain Beta1. Second, MRA for metabolites and GERD was conducted to obtain beta2. Third, MRA for inflammatory cytokines and GERD was performed to obtain Betal. Finally, the mediation effect analysis was calculated (Fig. 1). The mediation effect is determined as [Beta1*Beta2], and the direct effect as [Beta.all-Beta1*2].

## 3. Results

### 3.1. Bidirectional 2-sample MRA

#### 3.1.1. Causal effects of circulating inflammatory cytokines on GERD

**Figure 2.**
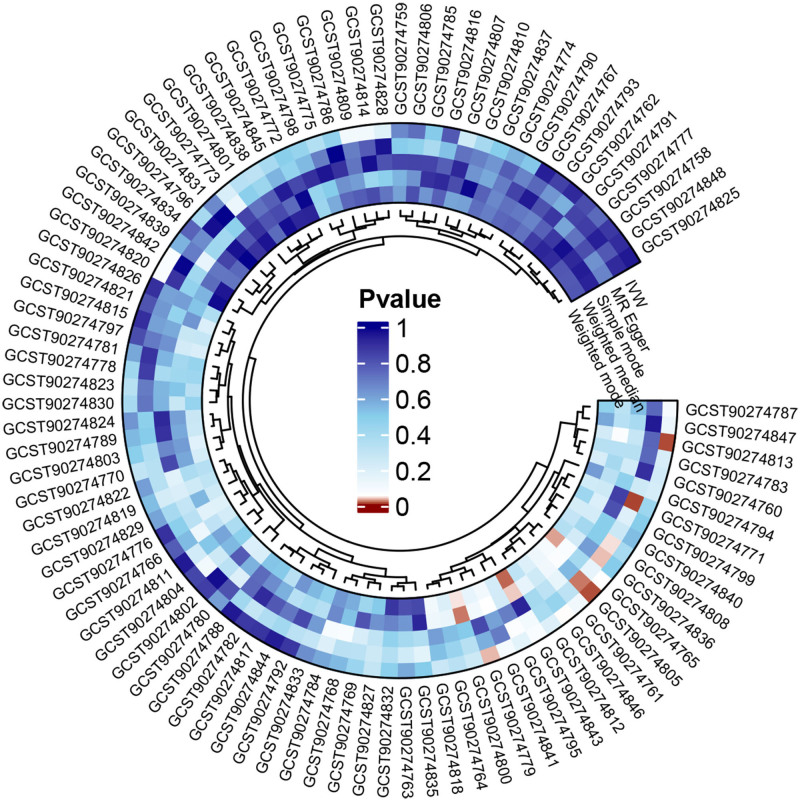
Circos heatmap of the causal relationships of 91 inflammatory cytokines on GERD (Forward MR analysis). GERD = gastroesophageal reflux disease, MR = Mendelian randomization.

**Figure 3.**
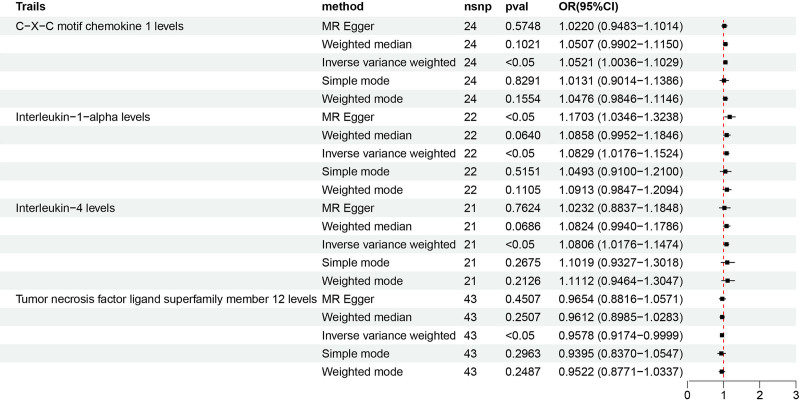
Forest plots of illustrating the causal correlation between 4 inflammatory cytokines and GERD (Forward MR analysis). GERD = gastroesophageal reflux disease, MR = Mendelian randomization.

**Figure 4.**
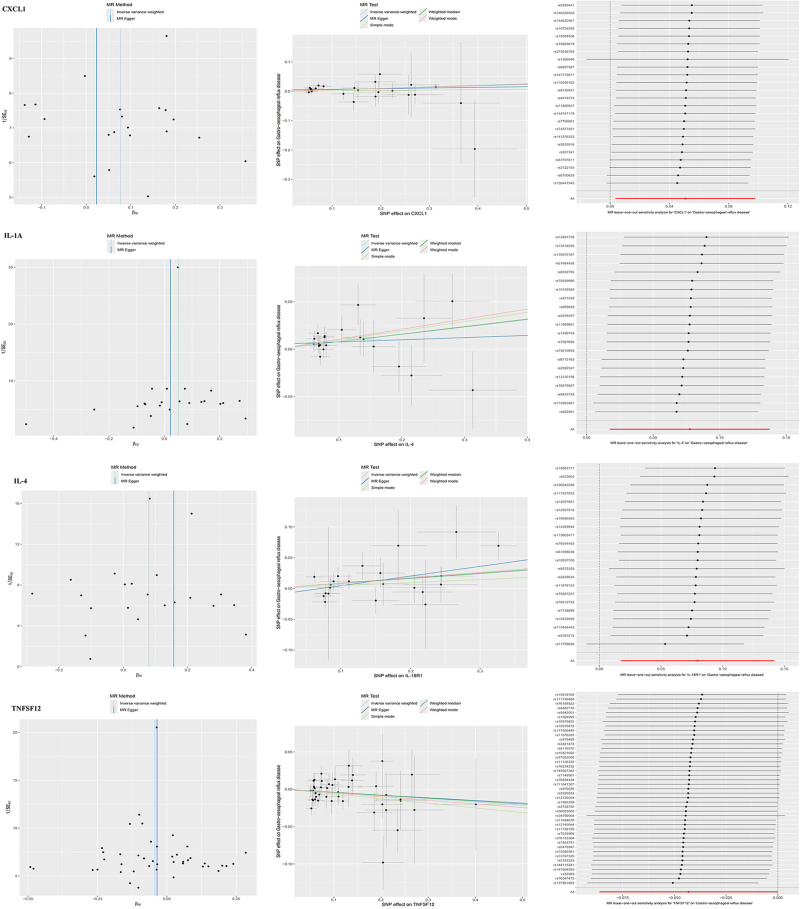
Scatter, funnel plots and LOO analysis of causal correlation between 4 inflammatory cytokines and GERD (Forward MR analysis). GERD = gastroesophageal reflux disease, LOO = leave-one-out, MR = Mendelian randomization.

After all the filtering procedures mentioned above, all involved SNPs became robust instruments with *F*-statistics ranging from 19.51 to 3549.33 (Table S1, Supplemental Digital Content, https://links.lww.com/MD/O970). The MRA results from different methods are presented in Table S4, Supplemental Digital Content, https://links.lww.com/MD/O970. Forward MRA outcomes indicated that the genetic susceptibility of 4 inflammatory cytokines associated and an increased GERD risk, while that of 87 other inflammatory cytokines showed no evidence of significant association with GERD risks (Fig. 2). Specifically, the preliminary results of IVW revealed 3 inflammatory cytokines with positive causal effects on GERD, including C-X-C motif chemokine 1 [CXCL1, OR = 1.052, 95% CI: 1.004–1.103, *P* = .035], interleukin-1-alpha [IL-1α, OR = 1.083, 95% CI: 1.018–1.152, *P* = .012], interleukin-4 [IL-4, OR = 1.081, 95% CI: 1.018–1.147, *P* = .011]. Contrarily, tumor necrosis factor ligand superfamily member 12 (TNFSF12) demonstrated negative causal effects on GERD [OR = 0.958, 95% CI: 0.9174–0.9999, *P* = .0493, Fig. 3]. The Cochran’s Q test for these 4 inflammatory factors did not reveal heterogeneity, no pleiotropy was observed for the MR-egger_intercept, and the results of the MR-PRESSO indicated no pleiotropy (Fig. 4). The results of leave-one-out analysis showed the robustness (Fig. 4). Apart from CXCL1, IL-1α, IL-4, and TNFSF12, the other 87 cytokines showed no association with GERD risk in the IVW primary MRA. (Table S4, Supplemental Digital Content, https://links.lww.com/MD/O970 and Fig. 3).

#### 3.1.2. Effect of GERD on inflammatory factors

**Figure 5.**
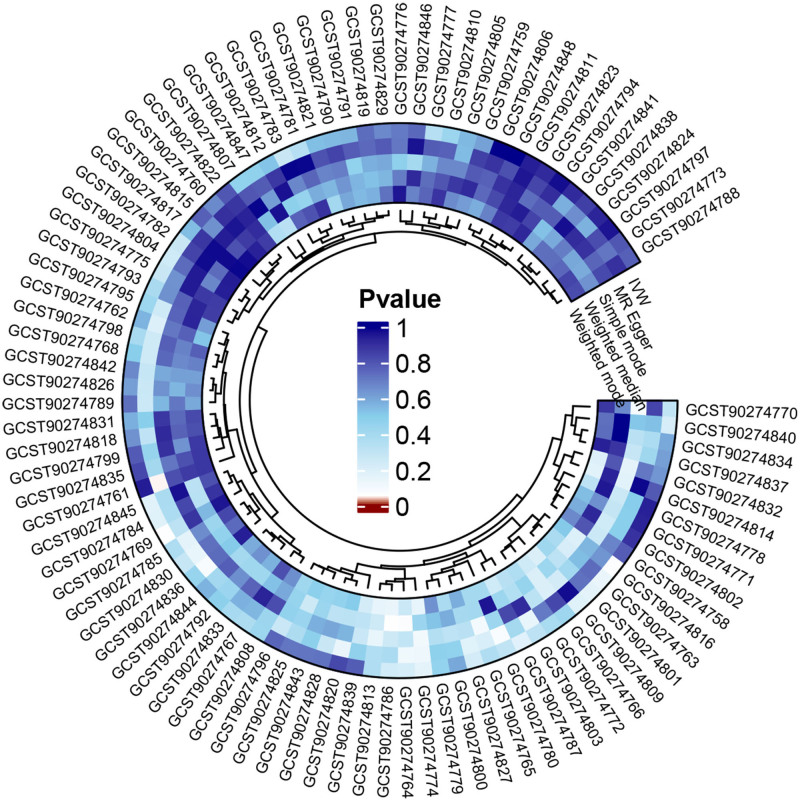
Circos heatmap of the causal association of GERD on 91 inflammatory cytokines (Reverse MR analysis). GERD = gastroesophageal reflux disease, MR = Mendelian randomization.

In reverse MR analysis, all selected SNPs were robust instruments, with *F*-statistics ranging from 19.58 to 31.00 (Table S2, Supplemental Digital Content, https://links.lww.com/MD/O970). IVW analysis indicated that the genetic susceptibility of GERD did not affect any circulating inflammatory cytokines-related traits (Table S5, Supplemental Digital Content, https://links.lww.com/MD/O970 and Fig. 5).

### 3.2. Two-step MRA

#### 3.2.1. Effect of inflammatory cytokines on metabolites

All selected SNPs related to metabolites were robust IVs. All of the *F*>10, as shown in Table S3, Supplemental Digital Content, https://links.lww.com/MD/O970. The causal effects of 8 inflammatory factors on 15 metabolites were identified via IVW (Table S6, Supplemental Digital Content, https://links.lww.com/MD/O970). As shown in Table 1, CXCL1 increased levels of carnitine C14:1 [OR = 1.093, 95% CI: 1.011–1.183, *P* = .026] and myristate [14:0, OR = 1.093, 95% CI: 1.032–1.206, *P* = .006]; IL-1α increased levels of (S)-a-amino-omega-caprolactam [OR = 1.096, 95% CI: 1.005–1.195，*P* = .039] and Cholate to phosphate ratio [OR = 1.139, 95% CI: 1.033–1.256，*P* = .009]; TNFSF12 increased level of X-17685 [OR = 1.094, 95% CI: 1.017–1.176, *P* = .016]. We found CXCL1 decreased Phenylpyruvate to 4-hydroxyphenylpyruvate ratio [OR = 0.902, 95% CI: 0.831–0.980, *P* = .015, Table 1]. Additionally, we also found that IL-4 decreased levels of 1-(1-enyl-oleoyl)-GPE [*P*-18:1, OR = 0.892, 95% CI: 0.800–0.996, *P* = .042], 4-methylguaiacol sulfate [OR = 0.871, 95% CI: 0.759–0.999, *P* = .048], X-12730 [OR = 0.879, 95% CI: 0.783–0.987, *P* = .029] and X-17685 [OR = 0.855, 95% CI: 0.762–0.958, *P* = .007, Table 1]. There was no significant heterogeneity detected by Cochan’s Q test, and MR-Egger intercept and MR-PRESSO tests did not show statistical significance (Table 1).

**Table 1:** Causal associations of inflammatory factor levels on circulating metabolites for IVW.

Inflammatory factors levels	Circulating metabolites levels	SNP (N)	MR results			Cochran’s Q statistic (*P*)	MR-egger intercept (*P*)
			OR	95% CI	*P*		
CCL4	X-10458	30	1.083	1.021–1.148	.008	.335	.732
	X-11632	30	1.072	1.004–1.144	.038	.099	.179
CCL1	Carnitine C14:1	23	1.093	1.011–1.183	.026	.638	.186
	Myristate (14:0)	23	1.116	1.032–1.206	.006	.498	.698
	Phenylpyruvate to 4-hydroxyphenylpyruvate ratio	23	0.902	0.831–0.980	.015	.861	.390
IL-1α	(S)-a-amino-omega-caprolactam	21	1.096	1.005–1.195	.039	.827	.779
	Cholate to phosphate ratio	21	1.139	1.033–1.256	.009	.819	.369
IL-4	1-(1-enyl-oleoyl)-GPE (*P*-18:1)	20	0.892	0.800–0.996	.042	.405	.507
	4-methylguaiacol sulfate	20	0.871	0.759–0.999	.048	.095	.830
	X-12730	20	0.879	0.783–0.987	.029	.739	.416
	X-17685	20	0.855	0.762–0.958	.007	.832	.462
IL-8	Glucuronide of C12H22O4 (1)	31	0.875	0.782–0.980	.021	.467	.581
MMP-1	7-methylxanthine	27	0.901	0.827–0.981	.016	.943	.989
OPG	X-13007	28	0.906	0.822–0.998	.047	.482	.940
TNFSF12	X-17685	44	1.094	1.017–1.176	.016	.631	.627

CCL1 = C-X-C motif chemokine 1, CCL4 = C-C motif chemokine 4, CI = confidence interval, IL-1α = interleukin-1-alpha; Interleukin-4, IL-8 = interleukin-8, IVW = inverse variance weighted, MMP-1 = matrix metalloproteinase-1, OPG = osteoprotegerin, OR = odds ratio.

#### 3.2.2. Effect of metabolites on GERD

Fifty seven metabolites significantly associated with GERD were identified, with no heterogeneity nor horizontal diversity (Table S7, Supplemental Digital Content, https://links.lww.com/MD/O970). Among these 57 robust causal relationships, 28 metabolites, demonstrated positive correlations with GERD. Conversely, 29 metabolites, negatively correlated with GERD. Notably, results of IVW revealed that levels of 3 metabolites, including Carnitine C14:1 [OR = 1.049, 95% CI: 1.006–1.094, *P* = .025], 4-methylguaiacol sulfate [OR = 1.094, 95% CI: 1.014–1.180, *P* = .021] and X-17685 [OR = 1.067, 95% CI: 1.014–1.123, *P* = .013] may increase risk of GERD; five metabolites, including (1-enyl-oleoyl)-GPE [*P*-18:1, OR = 0.946, 95% CI: 0.899–0.994, *P* = .029], X-12730 [OR = 0.920, 95% CI: 0.873–0.970, *P* = .002], myristate [14:0, OR = 0.933, 95% CI: 0.887–0.982, *P* = .008], cholate to phosphate ratio [OR = 0.936, 95% CI: 0.890–0.985, *P* = .010] and phenylpyruvate to 4-hydroxyphenylpyruvate ratio [OR = 0.956, 95% CI: 0.916–0.999, *P* = .043] may decrease the risk of GERD (Table 2). Meanwhile, these 8 metabolites were impacted by some specific inflammatory cytokines (Table 1). The sensitivity analyses revealed there was no heterogeneity nor pleiotropy, indicating robust and reliable results.

**Table 2:** Causal effects of circulating metabolites levels on GERD (*P* < .05).

Circulating metabolites	SNP (N)	MR results			Cochran’s Q statistic (*P*)	MR-egger intercept (*P*)
		OR	95% CI	*P*		
Xanthurenate level	25	1.068	1.022–1.115	.003	.601	.358
Docosahexaenoate DHA; 22:6n3 level	21	1.073	1.011–1.140	.021	.046	.962
Isovalerate (i5:0) level	17	1.070	1.012–1.131	.017	.859	.115
Cysteine s-sulfate level	16	1.085	1.023–1.151	.007	.959	.780
1-methylhistidine level	29	1.077	1.030–1.126	.001	.290	.662
N-acetylthreonine level	22	1.075	1.018–1.136	.010	.326	.352
1-stearoyl-gpc (18:0) level	31	1.044	1.006–1.084	.023	.362	.834
7-methylxanthine level	24	1.062	1.006–1.120	.030	.939	.784
1-stearoyl-GPE (18:0) level	40	1.046	1.013–1.080	.006	.984	.966
2-palmitoleoyl-GPC (16:1) level	25	0.934	0.887–0.984	.010	.080	.803
5alpha-pregnan-diol disulfate level	22	1.037	1.005–1.071	.025	.478	.859
N-acetylcarnosine level	28	0.960	0.929–0.992	.014	.074	.759
1-(1-enyl-oleoyl)-GPE (*P*-18:1) level	18	0.946	0.899–0.994	.029	.098	.248
1-oleoyl-GPG (18:1) level	25	0.969	0.940–0.998	.039	.568	.106
4-hydroxychlorothalonil level	23	0.961	0.924–0.999	.047	.569	.462
Carnitine C14:1 level	34	1.049	1.006–1.094	.025	.375	.538
4-methylguaiacol sulfate level	14	1.094	1.014–1.180	.021	.030	.958
4-methoxyphenol sulfate level	20	1.068	1.012–1.128	.017	.322	.769
Glycochenodeoxycholate 3-sulfate level	29	0.966	0.934–0.998	.038	.424	.366
N-palmitoyl-sphinganine (d18:0/16:0) level	23	1.046	1.000–1.094	.049	.662	.472
*P*-16:0/16:0 level	33	1.037	1.008–1.067	.013	.454	.561
Caffeic acid sulfate level	25	1.062	1.004–1.123	.035	.061	.065
Gamma-glutamyl-alpha-lysine level	31	0.964	0.934–0.994	.020	.528	.893
3-hydroxyoleoylcarnitine level	28	1.059	1.009–1.111	.019	.235	.012
3-CMPFP level	25	1.058	1.014–1.105	.010	.403	.958
Glucuronide of C12H22O4 (1) level	18	1.077	1.013–1.145	.018	.004	.964
(S)-a-amino-omega-caprolactam level	13	1.056	1.015–1.098	.007	.578	.952
3-ureidopropionate level	23	0.945	0.898–0.994	.030	.270	.805
Allantoin level	24	0.956	0.917–0.997	.034	.070	.465
3-hydroxy-3-methylglutarate level	21	0.945	0.896–0.996	.034	.369	.195
Taurodeoxycholate level	23	1.063	1.013–1.115	.013	.537	.603
Flavin adenine dinucleotide (FAD) level	24	1.027	1.002–1.053	.033	.760	.519
Histidine level	22	0.962	0.929–0.997	.033	.607	.161
S-1-pyrroline-5-carboxylate level	19	0.941	0.891–0.993	.028	.479	.264
Inosine 5’-monophosphate (IMP) level	22	1.079	1.032–1.127	.001	.050	.301
Myristate (14:0) level	23	0.933	0.887–0.982	.008	.507	.618
X-10458 level	24	0.942	0.893–0.994	.029	.068	.779
X-11632 level	25	0.933	0.876–0.994	.032	.003	.596
X-12193 level	27	0.954	0.911–1.000	.048	.365	.608
X-11880 level	18	0.946	0.897–0.998	.043	.401	.958
X-12730 level	18	0.920	0.873–0.970	.002	.291	.786
X-13007 level	28	0.943	0.908–0.980	.003	.415	.163
X-16580 level	18	1.063	1.005–1.123	.031	.420	.715
X-17685 level	21	1.067	1.014–1.123	.013	.809	.305
X-21364 level	32	1.054	1.013–1.096	.009	.975	.291
X-21310 level	21	0.946	0.900–0.995	.031	.410	.458
X-23678 level	19	0.954	0.913–0.998	.039	.460	.686
X-23659 level	28	1.040	1.008–1.072	.013	.684	.186
X-24801 level	34	0.960	0.926–0.996	.030	.363	.683
5-acetylamino-6-formylamino-3-methyluracil level	21	0.964	0.936–0.994	.018	.211	.184
2’-o-methyluridine level	17	1.029	1.004–1.055	.023	.315	.158
N-acetylputrescine to (N(1) + N(8))-acetylspermidine ratio	32	0.959	0.930–0.988	.007	.055	.047
Arginine to glutamate ratio	25	0.934	0.889–0.980	.006	.885	.205
Glycine to alanine ratio	20	0.963	0.934–0.993	.018	.530	.195
Phosphate to 2’-deoxyuridine ratio	33	1.050	1.007–1.095	.023	.002	.472
Dopamine 4-sulfate to dopamine 3-O-sulfate ratio	23	0.943	0.894–0.996	.034	.049	.777
3-phosphoglycerate to glycerate ratio	18	1.078	1.017–1.142	.011	.443	.613
Cholate to phosphate ratio	18	0.936	0.890–0.985	.010	.353	.993
ADP to glycerol 3-phosphate ratio	23	0.959	0.927–0.993	.017	.759	.512
ADP to glycerol ratio	24	1.048	1.011–1.087	.012	.240	.229
Histidine to phosphate ratio	29	1.041	1.002–1.082	.040	.213	.701
Cortisone to 4-cholesten-3-one ratio	32	0.968	0.939–0.997	.033	.135	.165
Phenylpyruvate to 4-hydroxyphenylpyruvate ratio	28	0.956	0.916–0.999	.043	.992	.897
Uridine to 2’-deoxyuridine ratio	27	1.053	1.006–1.103	.027	.232	.797
N-stearoyl-sphingosine (d18:1 to 18:0) to N-palmitoyl-sphinganine (d18:0 to 16:0) ratio	31	1.043	1.006–1.081	.024	.458	.017
Glutamate to pyruvate ratio	26	0.935	0.886–0.986	.014	.104	.545

ADP = adenosine diphosphate, FAD = flavin adenine dinucleotide, GERD = gastroesophageal reflux disease, IMP = inosine 5’-monophosphate.

#### 3.2.3. Mediation effect

Through the mediation analysis of the 1400 metabolites, 4 metabolites that may mediate the elevation of IL-4 levels and increased GERD risk were identified. In particular (Table S8, Supplemental Digital Content, https://links.lww.com/MD/O970), elevated IL-4 levels were linked to alterations in the levels of (1-enyl-oleoyl)-GPE (*P*-18:1), 4-methy lguaiacol sulfate, X-12730 and X-17685. Their mediation effects were 0.006 (95% CI: 0.00–0.015), −0.01 (95% CI: −0.023–0.004), 0.011 (95% CI: −0.001–0.023) and −0.010 (95% CI: −0.021–0.001) respectively. Although these metabolites demonstrated effects associated with changes in IL-4 levels, the mediation effects might not reach statistical significance (*P* > .05).

### 3.3. Sensitivity analysis

The results of sensitivity were displayed in Table S6–S7 and S9–S10, Supplemental Digital Content, https://links.lww.com/MD/O970. There was significant heterogeneity in several results. We used the random-effects model to analyze and the results of IVW did not substantially alter. Pleiotropy was found in several results of the MR-Egger intercept. Notably, after adjusting MR-PRESSO, the results remained consistent with the original findings.

## 4. Discussion

Data regarding inflammatory factors and metabolites were derived from GWAS, and a thorough analysis using bidirectional 2-sample and 2-step MRA approaches was employed to reveal the causal relationships between 91 inflammatory cytokines and GERD. Also, we conducted a mediation analysis to reveal whether the 1400 metabolites play key mediating roles in their causal relationships. Significant findings emerged, indicating that CXCL1, IL-1α, and IL-4 had a positive relation with GERD risk, while the relationship with TNFSF12 is negatively associated. Additionally, the genetic susceptibility to GERD has no causal effect on inflammatory cytokines levels. Mediation analysis suggested that (1-enyl-oleoyl)-GPE (*P*-18:1), 4-methyl guaiacol sulfate, X-12730, and X-17685 may be potential mediators of increased GERD risk mediated by elevated IL-4. Importantly, sensitivity analysis supported the robustness of the results. Our results indicate a substantial understanding of the functions of circulating inflammatory cytokines and metabolites in terms of GERD treatment.

The GERD pathogenesis is still not fully comprehended, but the irregularity in the generation and control of inflammatory cytokines plays a crucial role in its onset. Dysregulation of the inflammatory cytokines triggers the abnormal immune response in GERD patients, inducing inflammatory cascades. Studies have shown that GERD does not result from chemical esophagus damage caused by gastric content but rather by cytokine-mediated outcomes.^[20,21]^ CXCL1, a crucial chemokine in inflammation, primarily induces neutrophil recruitment to inflammatory sites.^[22]^ In the human body, CXCL1 acts as an important inflammatory response mediator, inducing neutrophil recruitment and participating in inflammatory responses, potentially leading to tissue damage.^[22]^ The findings indirectly support our results, suggesting that higher predicted levels of CXCL1 may increase the risk of GERD. This association may be due to the fact that CXCL1 recruits neutrophils, which produce reactive oxygen species and lipid peroxidation that are mainly involved in the progression of esophageal inflammation caused by gastroesophageal reflux.^[23]^

The positive MR analysis indicated that a relatively high predicted IL-1α level correlates with a heightened likelihood of GERD. Sensitivity analysis and pleiotropy analysis revealed the robustness of the causal relationship, and it does not show reverse causation. Interleukins (IL) are cytokines produced by a variety of cells. IL plays an important role in messaging, immune cell regulation, and inflammatory responses. IL-1α is a cytokine that triggers and enhances inflammatory responses.^[24]^ IL-1α is produced by endothelial and epithelial cells via pro-IL-1α processing.^[24]^ Exposure to pathogens or stressful stimuli dramatically increases IL-1α expression, which triggers an inflammatory response.^[25]^ The release of IL-1α is commonly associated with necrotic cell death resulting in inflammation. IL-1α binds to the IL-1 receptor type I to activate the IL-1 signaling pathway.^[24]^ Bile acids, deoxycholic acid, goose deoxycholic acid and pepsin are major components of gastric contents. Preclinical evidence suggests that these components can induce IL-1α secretion in bone marrow-derived dendritic cells in vitro.^[26]^ It has also been shown that inflammasome activators induce cellular secretion of IL-1α.^[27]^ Pro-IL-1α binds to mitochondrial cardiolipin in activated macrophages and enhances NLRP3 inflammatory vesicle activation.^[28]^ Clinical evidence shows that in patients with gastroesophageal reflux, pepsin can activate the NLRP3 inflammasome, and inhibiting the NLRP3 is able to control epithelial cell damage.^[29]^ In patients with pharyngeal reflux, an increased number of laryngeal epithelial and subepithelial IL-1α structures has been associated with inflammation, proliferation, and tissue remodeling.^[30]^ These evidences provide a possible explanation for our research findings.

IL-4 is primarily generated by CD4 + T cells once they are activated.^[31]^ IL-4 can act on a broad spectrum of cell types, encompassing hematopoietic precursor cells, macrophages, NK cells, stromal cells, and fibroblasts.^[32]^ Additionally, IL-4 is a symbolizing lymphocyte 2 cytokine, which is essential for symbolizing lymphocyte 2 cell differentiation.^[33]^ Certain theories suggest that the occurrence of GERD is due to T lymphocytes attracting chemical inducers from the basal layer to the mucosa.^[34]^ Previous research demonstrates that IL-4 plays a significant effect on the pathogenesis of GERD.^[35]^ This aligns with the results obtained in our research, specifically, the MR analysis result of the research confirms the role of IL-4 as one of the risk factors for GERD.

The MR results indicate TNFSF12 may decrease the risk of GERD. TNFSF12, aka TNF-like weak inducer of apoptosis, acts as a cytokine primarily expressed by monocytes and macrophages.^[36]^ TNFSF12 acts as a type II transmembrane protein, as well as a cleaved soluble molecule. The report indicated that in cases of GERD, human esophageal microvascular endothelial cells (HEMEC) react to acidic pH stress through the phosphatidylinositol 3-kinase (PI3K)/Akt pathway, and extended exposure to acidic pH leads to pronounced phosphorylation of Akt in HEMEC.^[37]^ Studies have shown that TNFSF12 protects cardiomyocytes from apoptosis in a manner reliant on the PI3K/AKT signaling pathway.^[38]^ These studies provide a possible explanation for our MR analysis results: TNFSF12 protects HEMEC from apoptosis via the PI3K/AKT pathway, thereby reducing the risk of GERD.

The MR analysis results demonstrated that CXCL1, IL-1α, and IL-4 are potential risk factors for GERD, while TNFSF12 is a potential protective factor for GERD, and there is no reverse causation of the relationship. However, no studies to date have directly revealed the link between inflammatory cytokines and GERD risk. Therefore, Future studies could conduct further randomized controlled trials to directly confirm the causal correlation between these inflammatory factors and GERD. Large-sample prospective studies could also be conducted to see how the association between these inflammatory factors and GERD develops over time and how this relationship manifests itself in different age groups and life stages. Based on the findings of this study, future intervention studies targeting these positive findings for inflammatory factors and GERD could be conducted to assess the impact of interventions for these conditions on patient prognosis.

Previous evidence has shown that the levels of GATA3, IL-18, TLR4, TNFSF12A, and CD68 are negatively associated with the mean esophageal pH;^[6]^ and that the IL-10 and IL-8 are associated with histological alterations in the esophageal lining.^[39]^ The levels of IL-8, serum IL-10, and TNFSF12-a grow by a degree in patients with GERD as disease progresses.^[40]^ Furthermore, clinical evidence suggests that serum interleukin-6, IL-8, TNFSF12-a-levels are highly valuable for assessing the severity of GERD. However, both positive and reverse MR analyses suggested that these circulating inflammatory cytokines have no significant causal relationship with GERD risk. The contradictory results may be attributed to potential confounding factors and racial differences. Given the biological functions of these inflammatory cytokines and the current bottlenecks in developing GERD treatments, further researches should be conducted to fully grasp the causal link between the inflammatory factors and GERD risk, with a view to help better manage GERD.

The mediation analysis results indicated that although changes in the levels of (1-enyl-oleoyl)-GPE (*P*-18:1), 4-methyl guaiacol sulfate, X-12730, and X-17685 are associated with the growth of IL-4 level, the evidence for their acting as potential mediators in the pathway between IL-4 and GERD risk is not sufficiently solid. This may result from inadequate sample size, small effect size, measurement, or other unconsidered confounding factors. Although the current analysis is not yet adequate to establish the significance of these metabolites as mediators, they may still contribute to the biological pathway between IL-4 and GERD risk. The possible mechanisms of these metabolites and their potential role in the management of GERD is needed to be investigated in further study.

The study has several strengths. While previous research has confirmed the causal correlation between specific inflammatory cytokines and GERD, disputes occur resulting from complex confounders and reverse causality. So far, no published study has been able to reveal these causal relationships based on reliable causal inference methods. Meanwhile, our MRA marks the first extensive and thorough study to delve into the correlations between 91 inflammatory factors and GERD. Meanwhile, we firstly performed a 2-step MR to reveal the mediation effects of 1400 blood metabolites at the gene prediction level. The study made a significant finding that CXCL1, IL-1α, and IL-4 are confirmed to correlate with a heightened risk of GERD, while the levels of TNFSF12 are linked to a reduced risk of GERD. In addition, the major advantage of the research is found in the MRA design and mediation analysis. Observational studies can be vulnerable to unidentified confounders and reverse causality, which are addressed by MRA. MRA investigates causal relationships via genetic IVs associated with the exposure and the outcome, thereby eliminating the impact of confounding variables, as well as reverse causality.^[8]^ Furthermore, we conducted rigorous outlier assessments and extensive sensitivity analysis to increase the reliability of the outcomes, thereby bolstering the trustworthiness of our discoveries.

There are constraints in the study. First, the GWAS data used in the research were gained from European ancestry, posing challenges in extrapolating the conclusions to other populations. Secondly, the use of aggregated data from GWAS precluded stratified analyses based on parameters. Finally, although pleiotropy tests and the MR-PRESSO method were employed to prevent confounding caused by pleiotropy, residual bias is difficult to avoid, as it’s a recognized limitation of MRA.

## 5. Conclusion

This research shows that increased levels of CXCL1, IL-1α, and IL-4 correlate with a heightened risk of GERD, while TNFSF12 is linked with a decreased risk, but the causal relationships are not bidirectional. Additionally, some circulating metabolites are associated with elevated IL-4 levels, but the evidence supporting their roles as potential mediators of the pathway between IL-4 and GERD risk is not yet sufficient. The findings of this study aid in enhancing the comprehension of the pathogenesis of GERD and underscore the possibility of treatments aimed at inflammatory cytokines.

## Acknowledgments

The authors would like to convey their heartfelt appreciation to the researchers generously sharing the GWAS data we utilized.

## Author contributions

**Conceptualization:** Sheng Xie.

**Data curation:** Chengning Yang, Guangwen Chen.

**Formal analysis:** Jieru Xie.

**Project administration:** Sheng Xie.

**Software:** Jinjing Tan.

** Writing – original draft:** Liqun Li, Lijian Liu.

**Writing – review & editing:** Xiaoyan Huang.

## Supplementary Material


